# Characterization of a cytochrome P450 that catalyzes the *O*-demethylation of lignin-derived benzoates

**DOI:** 10.1016/j.jbc.2024.107809

**Published:** 2024-09-21

**Authors:** Megan E. Wolf, Daniel J. Hinchen, John E. McGeehan, Lindsay D. Eltis

**Affiliations:** 1 Department of Microbiology and Immunology, Life Sciences Institute and Bioproducts Institute, The University of British Columbia, Vancouver, Canada; 2 Centre for Enzyme Innovation, School of Biological Sciences, University of Portsmouth, Portsmouth, UK

**Keywords:** *O*-demethylation, lignin, cytochrome P450, biocatalysis, crystal structure, heme enzyme

## Abstract

Cytochromes P450 (P450s) are a superfamily of heme-containing enzymes possessing a broad range of monooxygenase activities. One such activity is *O*-demethylation, an essential and rate-determining step in emerging strategies to valorize lignin that employ carbon–carbon bond cleavage. We recently identified PbdA, a P450 from *Rhodococcus jostii* RHA1, and PbdB, its cognate reductase, which catalyze the *O*-demethylation of *para*-methoxylated benzoates (*p*-MBAs) to initiate growth of RHA1 on these compounds. PbdA had the highest affinity (*K*_d_ = 3.8 ± 0.6 μM) and apparent specificity (*k*_cat_/*K*_M_ = 20,000 ± 3000 M^-1^ s^-1^) for *p-*MBA. The enzyme also *O*-demethylated two related lignin-derived aromatic compounds with remarkable efficiency: veratrate and isovanillate. PbdA also catalyzed the hydroxylation and dehydrogenation of *p-*ethylbenzoate even though RHA1 did not grow on this compound. Atomic-resolution structures of PbdA in complex with *p*-MBA, *p-*ethylbenzoate, and veratrate revealed a cluster of three residues that form hydrogen bonds with the substrates’ carboxylate: Ser87, Ser237, and Arg84. Substitution of these residues resulted in lower affinity and *O*-demethylation activity on *p*-MBA as well as increased affinity for the acetyl analog, *p-*methoxyacetophenone. The S87A and S237A variants of PbdA also catalyzed the *O*-demethylation of an aldehyde analog of *p*-MBA, *p*-methoxy-benzaldehyde, while the R84M variant did not, despite binding this compound with high affinity. These results suggest that Ser87, Ser237, and Arg84 are not only important determinants of specificity but also help to orientate that substrate correctly in the active site. This study facilitates the design of biocatalysts for lignin valorization.

The cytochrome P450 (P450) superfamily of heme-thiolate enzymes catalyzes critical reactions across all domains of life (1). Due in part to their remarkable range of substrates and reactivities, these enzymes are promising biocatalysts (2, 3). Bacterial P450s play a key role in the degradation of carbon compounds in the environment, facilitating their use as growth substrates. One class of such compounds is aromatic compounds resulting from the biological degradation of lignin, an abundant component of woody biomass (4). Indeed, bacterial P450s are one of only three enzyme families known to catalyze the *O*-demethylation of lignin-derived aromatic compounds (LDACs), a necessary step for their catabolism (5).

The ability of bacteria to catabolize LDACs has been exploited for the valorization, or upgrading, of lignin. Lignin is an abundant, renewable source of aromatic compounds, and therefore a potential replacement for fossil fuels in the production of industrial chemicals (6). However, lignin’s heterogeneity and recalcitrance are barriers to its conversion to higher-value compounds. One promising strategy for lignin valorization involves a tandem process in which lignin is first chemically fractionated into a mixture of aromatics (7, 8). The constitution of the mixture is dependent on the method of fractionation and the biomass source. For example, the reductive catalytic fractionation of corn stover lignin generates a mixture of alkylguaiacols and alkylsyringols (9). A more recently developed fractionation method involves a methylation protection step to increase the yield of monomers (10). When applied to pine biomass, this generates a mixture in which the primary monoaromatics are *p*-methoxylated: veratrate, veratraldehyde, and *para*-methoxybenzoate (*p*-MBA). In the second step of the tandem process, a microbial cell factory is then used to convert the mixture of LDACs to a single, platform chemical in high atom yield (11). One critical and rate-limiting reaction in this biocatalytic conversion is *O*-demethylation (12, 13, 14). *O*-Demethylation is especially critical for biocatalysts designed to convert mixtures of methoxylated LDACs. The characterization and optimization of P450s and other LDAC *O*-demethylases is therefore critical to biocatalyst design (15).

We recently characterized the growth of *Rhodococcus jostii* RHA1 (RHA1 hereafter) on a methylated lignin stream from pine and identified the enzymes responsible for the degradation of the major LDACs (16). PbdA, a P450, and its partner reductase, PbdB, catalyzed the *O*-demethylation of the LDACs *p*-MBA and veratrate (Fig. 1) to *p*-hydroxybenzoate and vanillate, respectively. PbdA is a member of the CYP199A family (17), which appears to have evolved to *O*-demethylate *p*-MBAs. For example, CYP199A4, the best characterized member of the family, also catalyzes the *O*-demethylation of *p*-MBA and veratrate (18). Other family members that catalyze one or both of these reactions are CYP199A2, A25, and A35 despite sharing as little as 51% amino acid sequence identity (19, 20, 21). CYP199A4 also catalyzes a number of other reactions, albeit less efficiently than *O*-demethylation. Thus, the enzyme hydroxylates and desaturates *p-*ethylbenzoate (*p-*EB, Fig. 1) at approximately half the rate as *p*-MBA *O*-demethylation (21). In addition, CYP199A4 catalyzes the *N*-demethylation and sulfoxidation of *para*-substituted benzoates, albeit at significantly lower rates than *O*-demethylation (22).

**Figure 1:**
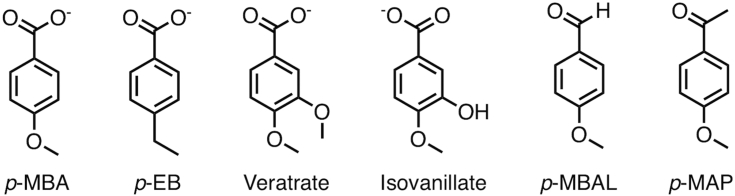
**Chemical structures of compounds used in this study.***p*-MBA, *p*-methoxybenzoate; *p*-EB, *p*-ethylbenzoate; *p*-MBAL, *p*-methoxybenzaldehyde; *p*-MAP, *p*-methoxyacetophenone.

Structural studies of CYP199A2 and A4, both from *Rhodopseudomonas* species, in the resting and substrate-bound states have revealed several structural elements of the CYP199A family (23). Notably, a substrate channel is in an “open” conformation in the resting state enzyme and is closed in the substrate-bound P450s. Two conserved serine and one arginine residues coordinate the carboxylate of the substrate and a collection of nonpolar residues interact with the aromatic ring. Finally, Phe185 plays an important in modulating hydroxylase and desaturase activity on alkylbenzoates: its substitution with smaller residues increased the distance of the ethyl chain from the heme iron, favoring hydrogen abstraction over the radical rebound mechanism which leads to hydroxylation (21).

Here, we describe the structure and function of PbdA characterized with its cognate reductase, PbdB. The yield and activity of PbdB were significantly improved by producing the protein in the native host with a codon-optimized construct and anaerobic purification. We characterized the substrate range and specificity of the P450 system. Guided by structural studies, we used mutagenesis to investigate the role of potential specificity-determining residues. The results are discussed with respect to the CYP199A subfamily of P450s as well as the development of biocatalysts for lignin valorization and other applications.

## Results

### Purification and substrate affinity of PbdA

**Figure 2:**
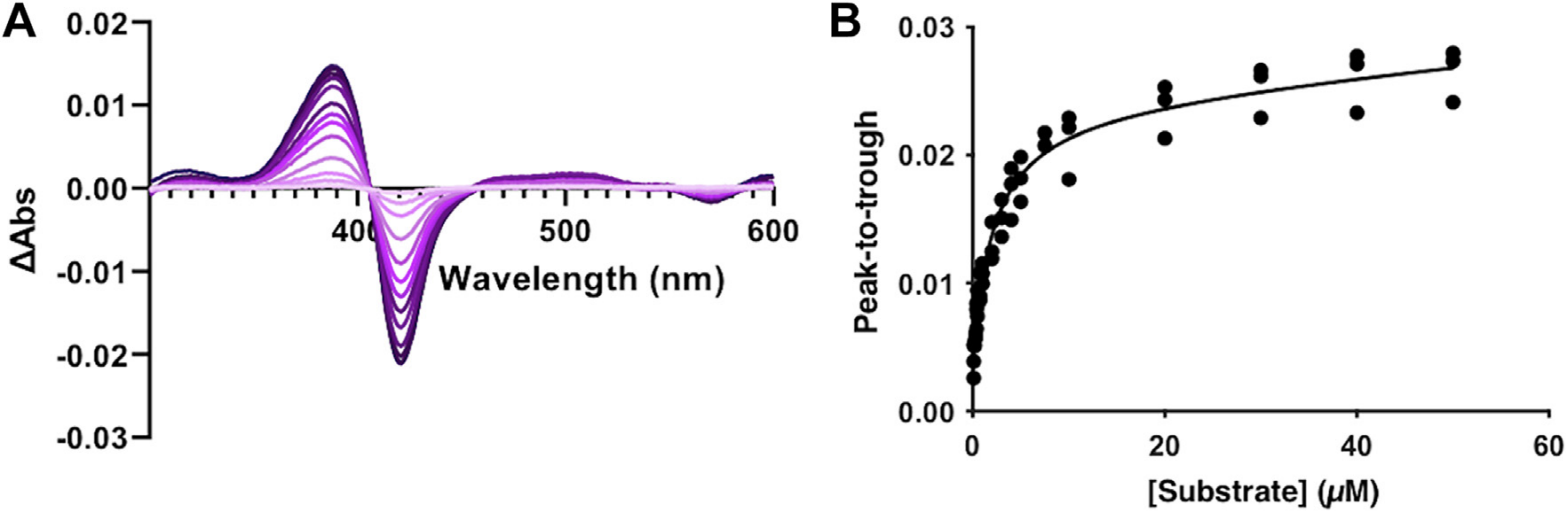
**Substrate binding of PbdA**. *A,* difference spectra of PbdA titrated with 1 to 50 μM *p*-MBA from the substrate-free enzyme. *Darker colors* indicate higher concentration of *p*-MBA. Spectra were collected after each addition of substrate to 1 μM enzyme in 20 mM Mops, *I* = 0.1 M, pH 7.2. *B,* binding isotherm of PbdA and *p*-MBA. The fitted curve represents a simple hyperbola. *p*-MBA, *para*-methoxybenzoate.

To characterize the activity of PbdA, we overproduced the enzyme in *Escherichia coli* and purified it to > 90% apparent homogeneity (Fig. S1*A*). As isolated, PbdA had an R_z_ of 0.66 (R_z_ = A_418_/A_280_). After reconstitution with heme, the R_z_ was 1.6 (Fig. S1*B*). This is similar to values reported for other CYP199As, which are between 1.5 and 2 (18, 20). After treatment with a reducing agent, PbdA bound CO as measured by the characteristic absorbance at 450 nm of the ferric-monoxide coordination complex (Fig. S1*C*). The affinity of PbdA for various LDACs was measured by the type I spectral shift upon titration of aromatic compound (Fig. 2). This shift occurs as the compound displaces a water coordinated to the heme iron, initiating a spin state transition in the latter from a low spin, hexacoordinate, to a high spin, pentacoordinate form. PbdA bound *p*-MBA, veratrate, and isovanillate with the highest affinity for *p*-MBA (Table 1). Moreover, the binding of *p*-MBA resulted in a near 100% transition of the heme iron to the high-spin form.

**Table 1:** Binding constants and apparent steady-state kinetic parameters of PbdA^a^

Substrate	K_*d*_	%High-spin^b^	K_*M*_	k_*cat*_	k_*cat*_*/*K_*M*_
	μM		μM	s^−1^	M^-1^ s^-1^
*p*-MBA	3.8 (0.6)	>99	18 (2)	0.37 (0.02)	20,000 (3000)
*p*-EB	6.0 (0.7)	72	12 (2)	0.14 (0.01)	11,000 (2000)
Veratrate	18 (3)	84	22 (5)	0.20 (0.02)	9000 (300)
Isovanillate	90 (20)	94	30 (10)	0.15 (0.03)	5000 (30)

*p-*EB, *p-*ethylbenzoate; *p*-MBA, *para*-methoxybenzoate.

a Experiments were performed using air-saturated Mops (*I* = 0.1 M), pH 7.2, at 25 °C. Because the steady-state conditions were not saturating with respect to PbdB concentration, the parameters are apparent. Parameters were calculated using a minimum of 15 data points at various substrate concentrations. SD is shown in parentheses.

b Percent of heme iron in high-spin state.

### Activity of PbdAB

To characterize the activity of the system, we produced PbdB, the cognate reductase, by overexpression in RHA1. Initial attempts to produce PbdB resulted in low yields, even in its native host. Inspection of its coding sequence revealed ten rare codons (<0.5% use) in the first 20 codons. As shown (24), substitution of these with frequently used codons resulted in a >20-fold increase in protein production. We purified the enzyme to >90% homogeneity anaerobically (24). Handling PbdB anaerobically maximized reductase activity as evaluated using a cytochrome *c* reduction assay. Anaerobically prepared PbdB also contained near stoichiometric amounts of flavin and iron-sulfur cofactors (24). PbdA, when combined with PbdB, O_2_, and NADH efficiently catalyzed the *p-O*-demethylation of *p*-MBA, veratrate, and isovanillate (Fig. S2).

We next tested the coupling of the reaction and its dependence on the ratio of the two components and ionic strength (Fig. S3). The rate of *p*-MBA *O*-demethylation increased with concentration of reductase in the reaction mixture. However, the coupling decreased above a molar ratio of 1:1 of PbdA to PbdB (Fig. S3*A*). Coupling and reaction rate were maximal around 100 to 125 mM ionic strength (Fig. S3*B*). We then measured coupling under optimum conditions for other substrates. The coupling of the *O*-demethylation of veratrate and isovanillate to NADH oxidation were both approximately 75% coupled (Table 2). Finally, we measured the apparent steady-state kinetic parameters of the PbdA reaction under conditions of maximal coupling (Fig. S4). Under these conditions, the enzyme had the highest apparent specificity for *p-*MBA with decreasing specificity for veratrate and isovanillate (Table 1).

**Table 2:** Coupling of NADH oxidation and aromatic substrate turnover of PbdAB^a^

Substrate	NADH depletion rate	Rate of aromatic turnover	Coupling
	U mg P450^-1^	U mg P450^-1^	%
*p*-MBA	2700 (100)	2700 (200)	100 (3)
*p*-EB	830 (80)	900 (100)	110 (7)
Veratrate	2830 (50)	2030 (60)	72 (3)
Isovanillate	2200 (200)	1600 (100)	75.3 (0.1)

*p-*EB, *p-*ethylbenzoate; *p*-MBA, *para*-methoxybenzoate.

a The rate of aromatic turnover was based on the appearance of *O*-demethylated product for *p*-MBA, veratrate, and isovanillate and the disappearance of *p*-EB. Standard deviation is shown in parentheses.

### Hydroxylation and desaturation activities of PbdA

In addition to *O*-demethylation, CYP199A4 catalyzes other reactions, including the desaturation and hydroxylation of *p*-EB (22). To investigate how widespread these activities are in the CYP199A subfamily, we characterized the ability of PbdA to bind and transform *p*-EB. PbdA bound *p*-EB with close to the same affinity as *p*-MBA, its preferred substrate (Table 1). The transformation of *p-*EB by PbdAB was remarkably well-coupled to NADH consumption (Table 2). Moreover, the apparent *k*_cat_/*K*_M_ of PbdAB for *p*-EB was within 50% that for p-MBA (Table 1). Finally, we analyzed the products of *p*-EB oxidation by RHA1 resting cells induced to express the PbdAB system. Both desaturation and hydroxylation products were detected, along with acetyl and epoxide derivatives presumably produced through secondary oxidation of the desaturation product (Fig. S5). Despite the efficient oxidation of *p*-EB by PbdAB, RHA1 did not grow on this compound (Fig. S6).

### Structure of PbdA

**Figure 3:**
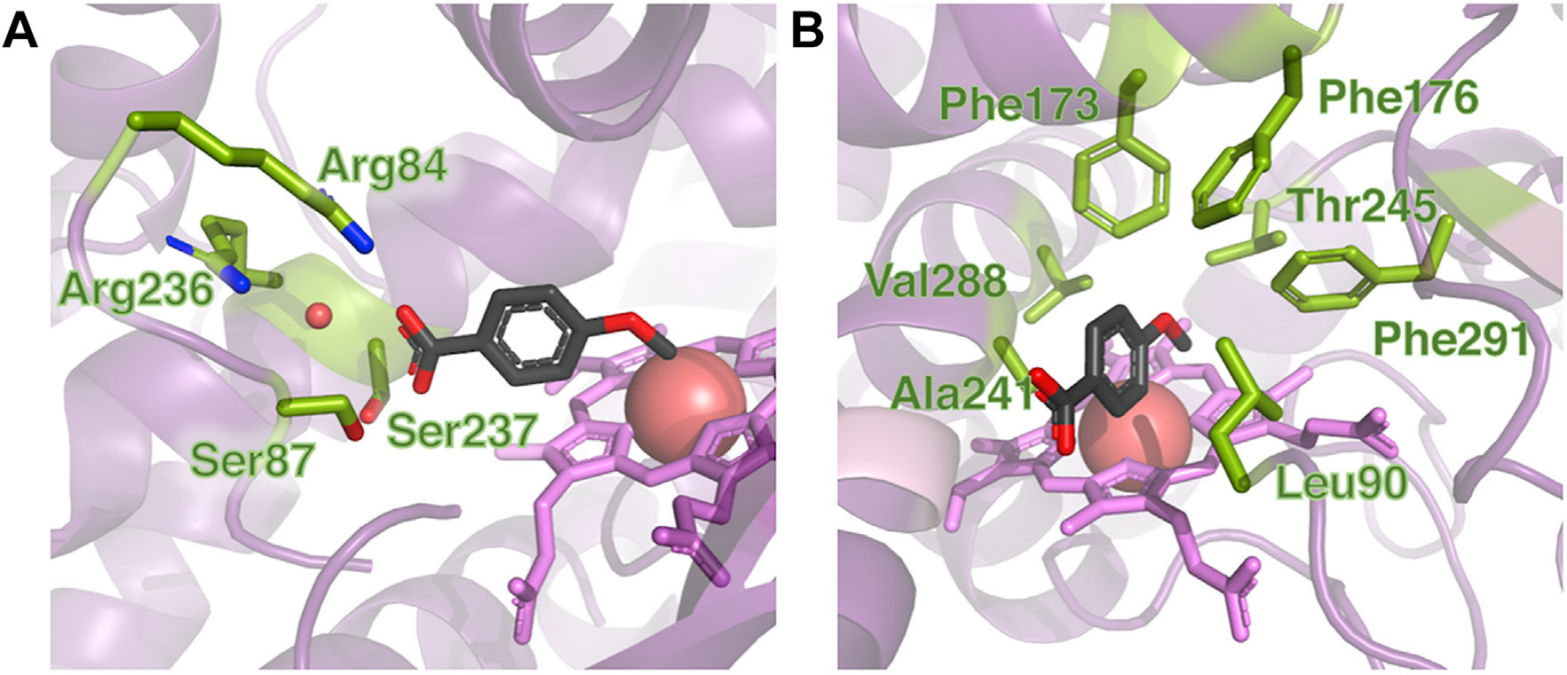
**Active site of PbdA.** Residues important for (*A*) carboxylate coordination and (*B*) hydrophobic interaction with the substrate are labeled. The heme and *p*-methoxybenzoate carbon atoms are shown in *magenta* and *black*, respectively. Structure shown is 9G9Q.

We obtained high-resolution crystal structures of PbdA in complex with *p*-MBA, veratrate, and *p*-EB at resolutions between 1.65 and 2.03 Å. Collection and refinement data are shown in Table S4. The structure of the three complexes are very similar to each other (RMSD of 0.176–0.683 Å of αC) as well as to the other CYP199As (*e.g*., RMSD of PbdA·*p-*MBA with CYP199A4·*p-*MBA and CYP199A2·*p-*MBA is 0.989 Å and 0.883 Å, respectively). Similar to the substrate-bound structures of CYP199A2 and CYP1994, all three structures were in a closed conformation, with the F and G helices occluding the substrate access channel. Substrate orientation is maintained by four hydrogen bonds to the carboxylate, one from ordered water and three from active site residues Arg84, Ser87, and Ser237. The ordered water is localized by hydrogen bonding by heteroatoms in Arg236 and Ser237 side chains (Fig. 3*A*). The aromatic ring of the substrate is stabilized by hydrophobic interactions with Leu90 and Ala241. Residues Phe173, Phe176, Phe291, Ala241, Thr245, and Val288 form a hydrophobic pocket for the aromatic ligand (Fig. 3*B*). These residues are all conserved in CYP199A4 (Fig. S7). The heme iron was positioned 4.1 Å from the methoxy carbon, a similar distance to reported Fe-C bond distance in P450_cam_ (25).

### Contribution of carboxylate-binding residues to substrate recognition

To interrogate the importance of the carboxylate-binding residues as specificity determinants, we substituted Arg84, Ser87, and Ser237 with residues of similar size that were unable to form hydrogen bonds with the substrate and assayed the binding and activity of the resulting variants for *p*-MBA. The R84M, S87A, and S237A variants all had approximately an order of magnitude lower affinity for *p*-MBA than WT PbdA, with the substitution at Ser87 having the greatest effect (Table 3). The substitution at Ser87 also had the greatest effect on specific activity for *p*-MBA, reducing it by approximately 3-fold (Table 3). Finally, a double variant in which both serine residues were substituted had an even lower affinity and specific activity, with the relative *K*_d_ values indicating that the two residues have partially additive effects on substrate binding.

**Table 3:** Affinity and specific activity of PbdA and its variants for *p*-MAP and *p*-MBAL^a^

Substrate	Variant	K_*d*_	%High-spin^b^	Specific activity
		μM		U μg P450^-1^
*p-*methoxybenzoate	WT	3.8 (0.6)	>99	2700 (100)
	R84M	36 (6)	56	1100 (200)
	S87A	50 (10)	81	800 (200)
	S237A	29 (5)	>99	2600 (400)
	S87A/S237A	70 (10)	44	180 (10)
*p*-methoxyacetophenone	WT	37 (9)	2.8	N.D.
	R84M	8 (1)	33	N.D.
	S87A	14 (2)	19	N.D.
	S237A	12 (1)	36	N.D.
	S87A/S237A	8 (1)	24	N.D.
*p*-methoxybenzaldehyde	WT	11 (1)	7.9	N.D.
	R84M	7 (1)	41	N.D.
	S87A	16 (3)	34	80 (30)
	S237A	12 (3)	23	27 (2)
	S87A/S237A	8 (2)	24	180 (20)

a SD is shown in brackets. N.D., not detected. Coupling data are provided in Table S5.

b Percent of heme iron in high-spin state.

To further investigate the contributions of the three carboxylate-binding residues, we tested the affinity and activity of the variants on two substrates analogs lacking the carboxylate: *p*-methoxyacetophenone (*p*-MAP) and *p*-methoxybenzaldehyde (*p*-MBAL) (Fig. 1). PbdA bound both compounds with relatively high affinity (Table 3). For example, replacing the substrate’s carboxylate with an aldehyde had a lesser effect on the *K*_d_ than did substitution of any of the carboxylate-binding residues. Although the enzyme had a relatively high affinity for these compounds, they induced a relatively low percentage transition of the heme iron to the high-spin state. Moreover, PbdA did not detectably catalyze the *O*-demethylation of either compound. However, both compounds stimulated futile NADH consumption by the WT enzyme (Table S5). All tested variants had increased affinity for *p-*MAP, but none were active on this substrate. The serine variants synergistically increased affinity for *p*-MAP, and the arginine variant had the same affinity as the double variant. A similar phenomenon was observed for the affinity of the enzyme for *p*-MBAL. In contrast to *p*-MAP, the serine variants catalyzed the *O*-demethylation of *p*-MBAL and the substitutions were synergistic with respect to this activity. Additionally, *p*-MBAL *O*-demethylation was well-coupled to NADH consumption in the double variant. Despite having the highest affinity for *p*-MBAL, the arginine variant did not turn it over. However, as with the other compounds that were not *O*-demethylated, NADH was consumed.

## Discussion

In this study, we demonstrated that the P450-reductase pair, PbdAB, catalyzes the efficient *O*-demethylation of three related LDACs: *p*-MBA, veratrate, and isovanillate. This is consistent with our previous findings that PbdAB is essential in the catabolism of these three compounds by RHA1 (16). The high affinity and specificity of PbdA for *p*-MBA is consistent with what has been reported for two members of CYP199A subfamily, A2, and A4, (18). However, PbdA differs from CYP199A2 and CYP199A4 with respect to its relative affinity and activity for veratrate. Thus, CYP199A2 and CYP199A4 bound *p*-MBA with three orders of magnitude higher affinity than veratrate. In contrast, PbdA bound *p*-MBA with approximately only five times higher affinity than veratrate. Moreover, CYP199A2 did not detectibly turn over veratrate, while PbdA did so at half the rate of *p*-MBA. This was somewhat unexpected given that the active site residues of CYP199A2/4 and PbdA are remarkably conserved. However, Ala174 (PbdA numbering) is a notable exception, corresponding to valine in CYP199A2 and CYP199A4 (Fig. S7). The increased side chain size could cause steric clash between the 3-methoxy group and the side chain (Fig. S8). Mutagenesis experiments could provide valuable insight into the effect of this residue. As veratrate is a major monoaromatic component of methylated lignin streams (10), high activity on veratrate would be advantageous for lignin-valorizing biocatalysts.

PbdAB also efficiently catalyzed the hydroxylation and desaturation of *p*-EB, as reported in CYP199A4 (21). Nevertheless, RHA1 did not grow on *p*-EB (Fig. S6). Indeed, a naturally occurring catabolic pathway for *p*-EB has yet to be reported. However, derivatives of *Pseudomonas* sp. B13 were evolved to utilize *p*-EB as a sole substrate *via meta*-cleavage, overcoming both regulation and enzyme inhibition, both of which are bottlenecks in its degradation by the WT strain (26). It is therefore likely that the high affinity and specificity of PbdA for *p*-EB reflect its chemical similarity to *p*-MBA and this is not a physiologically relevant reaction.

Structural studies of PbdA, coupled with substitution of active site residues, identified Arg84, Ser87, and Ser237 as specificity determinants through their respective interactions with the substrate carboxylate. This binding motif is conserved in the CYP199A family. Substitution of either Arg84 or Ser87 disrupted binding and activity significantly more than substitution of Ser237 (Table 3). This may reflect the fact that the former are coplanar while Ser237 is on a distal helix. Substitution of both serines in the double variant was most deleterious for binding and turnover of *p-*MBA. Indeed, S87A/S237A had the least negative binding energy, ΔG°, of any tested mutant (Table S6). Moreover, the ΔΔG° of the double variant *versus* WT PbdA was partially additive with respect to the two single variants, indicating additional stabilization mechanisms which compensate for the loss of hydrogen bonding to the carboxylate. Substitution of the Arg84 had a lower effect on binding energy than the substitution of both serines, possibly due to compensation by hydrogen bonding from the ordered water coordinated to Arg 236 (Fig. 2*A*).

The interaction of PbdA with substrate analogs further reveals the importance of interactions with the carboxylate in productively orientating the substrate for efficient *O*-demethylation. Thus, PbdA did not detectably *O*-demethylate *p*-MAP or *p*-MBAL despite binding both compounds with high affinity and consuming NADH in their presence. This is consistent with neither compound effectively displacing the heme iron-coordinated water molecule, as indicated by the spin state transition (Table 3). Although CYP199A4 catalyzed the *O*-demethylation of *p*-MAP and *p*-MBAL, it was at 0.25 and 0.1% the rate, respectively, of *p*-MBA (27). Interestingly, the S87A and S237A variants *O*-demethylated *p*-MBAL, although the arginine variant did not, despite having a relatively high affinity for this compound and *p*-MBAL inducing a relatively high degree of spin state transition. In the double variant, the *O*-demethylation of *p*-MBAL was remarkably well-coupled to NADH consumption (Table S5). None of the variants detectably turned over *p*-MAP, although this entire compound induced NADH consumption in all of them. This lack of turnover suggests that proper substrate orientation mediated by hydrogen bonding to the carboxylate is necessary for catalysis. Structural studies of PbdA and select variants in complex with substrate analogs might elucidate the underlying causes for the lack of catalysis.

This comprehensive structural and functional characterization of the P450 *O*-demethylase PbdA highlights its potential as a biocatalyst for the transformation of lignin streams. More specifically, advances in lignin processing are generating mixtures of novel methylated LDACs. Understanding the binding and turnover of methylated LDACs by PbdA facilitates the latter’s engineering toward biocatalytically upgrading such lignin streams.

## Experimental procedures

### Chemicals and reagents

All reagents were of analytical grade unless otherwise noted. Reagents were obtained from Sigma-Aldrich and Thermo Fisher Scientific. Media were prepared using water purified on a Barnstead NANOpure UV apparatus to a resistivity of greater than 16 MΩ/cm.

### DNA manipulation

Bacterial strains, plasmids, and primers used in this study are listed in Tables S1-S3, respectively. DNA was propagated, purified, and manipulated using standard procedures (28). The pET28a*_pbdA* protein expression plasmid was synthesized from TWIST Biosciences. The pTipQC1_*pbdB* was constructed by ordered the synthetic gene from TWIST Biosciences with overlaps appropriate for Gibson cloning into the pTip_QC1 plasmid, and assembling the plasmid *via* Gibson assembly. pET28a_*pbdA_S87A* was constructed by the Q5 mutagenesis kit (New England Biolabs) using the appropriate primers. pET28a_*pbdA_S237A,* pET28a_*pbdA_S87A/S237A, and* pET28a_*pbdA_R84M* were constructed by *via* the phosphorylated primers method using the appropriate primers and pET28a_*pbdA* and pET28a_*pbdA_S87A* as templates.

### Protein production and purification

PbdA and variants were produced heterologously as an N-terminal hexaHis-tagged (Ht-) protein in *E. coli* BL-21 λ (DE3) containing pET28a_*pbdA* and derivatives thereof. Freshly transformed cells were grown at 37 °C at 200 rpm in LB supplemented with 50 μg ml^−1^ of kanamycin until the culture reached an OD_600_ (optical density at 600 nm) of approximately 0.6. Expression of PbdA was induced with 0.5 mM isopropyl β-D-thiogalactopyranoside, and the cells were incubated at 30 °C for an additional 16 h. Cells were harvested by centrifugation. Cells collected from 1 L of culture were suspended in 20 ml of 20 mM Tris-Cl, 300 mM NaCl, 10 mM imidazole, 10% glycerol, pH 8 and lysed at 4 °C using an EmulsiFlex-C5 homogenizer (Avestin). Cellular debris was removed by centrifugation. Ht-PbdA was purified from the cell extract using immobilized metal affinity chromatography (HisPur Ni-NTA Resin, Thermo Fisher Scientific) according to the manufacturer’s protocol. Ht-PbdA was reconstituted with heme by dropwise addition of 3 M equivalents of 50 mM hemin in 0.1 M NaOH while stirring. The hexaHis tag was removed incubating Ht-PbdA with thrombin protease (50:1 M ratio) and dialyzing the mixture overnight against 20 mM Tris, pH 8.0, 10% glycerol at 4 °C. The protease-cleaved and uncleaved Ht-PbdA were separated by passing the dialyzed mixture over the Ni resin. PbdA was further purified using a MonoQ 10/100 Gl column and an ÄKTA Purifier (GE Healthcare). The protein was eluted with a linear gradient from 0 to 1 M NaCl in 120 ml of 20 mM Tris, pH 8.0. Fractions containing PbdA were pooled, dialyzed into 20 mM Tris, 10% glycerol pH 8.0 concentrated to approximately 10 mg mL^-1^ flash-frozen as beads, and stored at −80 °C.

The purification of PbdB is described in detail elsewhere (24). Briefly, PbdB was produced in RHA1 as an N-terminal Ht-protein in RHA1 containing pTipQC1_*pbdB.* Freshly transformed cells were grown at 30 °C at 200 rpm in LB supplemented with 35 μg ml^−1^ of chloramphenicol until the culture reached an OD_600_ of approximately 0.6. Expression of PbdA was induced with 10 μg mL^-1^ of thiostreptone, and the cells were incubated at 20 °C for an additional 48 h. Cells were harvested by centrifugation and disrupted by bead-beating. Protein was isolated by immobilized metal affinity chromatography (HisPur Ni-NTA Resin, Thermo Fisher Scientific) in an anaerobic chamber to maintain low-oxygen atmosphere. Fractions containing PbdB were pooled, buffer exchanged into 20 mM Tris, pH 8.0, 10% glycerol using a stirred-cell concentrator to approximately 10 mg ml^-1^, flash-frozen as beads, and stored at −80 °C.

### Crystallography

Purified protein of PbdA was concentrated to approximately 10 mg/ml for crystallization trials. Crystallization conditions were identified using the Mosquito crystal liquid handling robot (SPT Labtech) in SWISSCI 3-lens low profile plates, using various sparse matrix screens. Crystal hits were then optimized in hanging drop trays with best crystals grown in 0.8 M sodium phosphate monobasic, 0.8 M potassium phosphate, 0.1 Hepes at pH 7.5, and between 10 and 20 mM of various substrates, at 20 °C. One round of crystal seeding was required to produce crystals of diffraction quality. Crystals were cryoprotected in crystallization buffer containing 25% glycerol and directly cryocooled in liquid nitrogen. Diffraction data were collected at beamline I03 at the Diamond Light Source. The structure was solved by molecular replacement with Phaser (29) or MolRep (30), using Protein Data Bank: 4DNJ as the search model. Model building was performed in *Coot* (31), and the structure was refined with Refmac5 (32). MolProbity (33) was used to evaluate the final model, and PyMOL (pymol.org, Schrödinger, LLC) was used for protein model visualization. Structural figures were generated in PyMOL. RMSD values were calculated using the combinatorial extension (CE) alignment plugin in PyMOL (34). Structures have been deposited into the Protein Data Bank and are available under the accession codes: 9G9Q, 9G9R, and 9G9S.

### Protein analysis

UV-visible spectroscopy was conducted with a Cary 60 spectrophotometer equipped with a thermostatted cuvette holder at 25 °C. Scans of purified proteins diluted in 20 mM Mops, *I* = 0.1 M, pH 7.2 were taken between 250 and 700 nm. Heme concentration was calculated as described previously (35). Carbon monoxide binding was determined by reduction of PbdA diluted in 20 mM Mops, *I* = 0.1 M, pH 7.2 as above by a few grains of sodium dithionate. CO was bubbled into the reduced enzyme for approximately 60 s at a rate of one bubble per second. Dissociation constants (*K*_d_) were determined by titration of ligands diluted into enzyme buffer from 1 M stocks prepared in dimethyl sulfoxide into PbdA diluted in 20 mM Mops, *I* = 0.1 M, pH 7.2. Spectra take upon addition of substrate were subtracted from the spectrum of the enzyme, and peak-to-trough distance was plotted by ligand concentration. *K*_d_ values were determined by fitting a hyperbolic equation to the data as described previously (23, 36). The proportion of high-spin heme iron was determined using the approximate peak-to-trough extinction coefficient (ε ≈ 100 mM^−1^ cm^−1^) (36).

### Activity assays

For activity measurements 0.25 to 1 μM of PbdA and PbdB were combined in a 200 μl cuvette in 20 mM Mops, pH 7.2 with substrate diluted in the same buffer. NADH was added to 350 μM to initiate the reaction. For HPLC analysis, enzymatic reactions were acidified by adding acetic acid to 10% final concentration, centrifuged (16,000×*g* for 10 min), and filtered through a 0.2 μm polytetrafluoroethylene membrane. Samples were run over a Luna 5 μm C18(2) 100 Å 150 × 3 mm column (Phenomenex) at 0.7 ml min^−1^ by a Waters 2695 separation HPLC module. Samples were eluted with a 16.8 ml linear gradient from 1% methanol plus 0.1% formic acid in water to 100% methanol plus 0.1% formic acid and monitored at 280 nm with a Waters 2996 photodiode array detector. Concentrations were determined by interpolation on a standard curve of 0 to 1 mM of authentic standard. NADH concentration was measured by absorbance at 340 nm, ε = 6.22 mM^-1^ cm^-1^. Coupling was calculated from the ratio of rate of aromatic turnover, as determined by HPLC, and the rate of NADH oxidation, determined spectroscopically. Buffer ionic strength was adjusted by addition of NaCl, as appropriate. Apparent steady-state kinetic parameters were calculated by fitting Michaelis–Menten equations to initial velocity of reactions at various concentrations of aromatic substrate (LEONORA).

### RHA1 growth

RHA1 was grown in M9 media supplemented with a mixture trace metals (M9G) (37) and growth substrate. Growth substrates were solubilized in dimethyl sulfoxide and added to the media. For the growth on *p*-EB experiment, RHA1 was grown in 1 ml M9G supplemented with 5 mM benzoate for 1 day shaking at 30 °C. The cells were spun harvested and washed twice with M9G and added to 3 ml M9G containing *p*-EB or benzoate at a final OD_600_ of 0.05. OD_600_ of 150 μl culture in a 96-well plate was measured by a TECAN Spark microplate reader. For the resting cell transformation of *p*-EB, RHA1 was grown in 500 ml of M9G supplemented with 5 mM veratrate. When OD_600_ reached 0.6, cells were harvested, washed twice in M9G and suspended in 50 ml of M9G 1 mM *p*-EB. After 4 h of incubation, cell supernatant was collected for LC-MS analysis.

### LC/MS analysis of p-EB

LC-MS analyses were performed using an Agilent 1290 Infinity II UHPLC in line with an Agilent 6546 qTOF equipped with a dual AJS ESI source operating in positive ionization mode. Sample (2 μl) was injected onto a Zorbax Eclipse Plus C18 Rapid Resolution HD, 2.1 × 100 mm 1.8 μm column, and run with a 20 min linear gradient from 5 to 100% solvent B at 0.45 ml min^−1^. Solvent A was 0.1% formic acid in water, and solvent B was 0.1% formic acid in methanol. Mass spectrometer parameters were as follows: capillary voltage, 3500 V; nozzle voltage, 500 V; drying gas temp, 300 °C; drying gas flow rate, 10 L min^−1^; sheath gas temperature, 350 °C; sheath gas flow rate, 12 L min^−1^; nebulizer pressure, 45 psi; fragmentor voltage, 100 V. Data were collected using MassHunter Workstation LC/MS Data Acquisition version 10.1 and analyzed using MassHunter Workstation Qualitative Analysis version 10.0 (Agilent).

## Data availability

X-ray data of PbdA bound to *p*-MBA, veratrate and *p*-EB are deposited in the Protein Data Base under codes 9G9Q, 9G9R, and 9G9S, respectively.

## Supporting information

This article contains supporting information (21, 24, 38, 39, 40).

## Conflicts of interest

The authors declare that they have no conflicts of interest with the contents of this article.
