# Supplementary material for: Characterization of a cytochrome P450 that catalyzes the O-demethylation of lignin-derived benzoates
Source: J Biol Chem. 2024 Sep 21;300(11):107809. doi: 10.1016/j.jbc.2024.107809 (PMC11530827; doi:10.1016/j.jbc.2024.107809)
Supplement: Supplemental Figures and Tables [file mmc1.docx]

FIGURES

**Figure S1: Purification and reconstitution of PbdA.** (A) UV-Vis spectra of purified PbdA in 20 mM Tris-Cl, pH 8, 10% glycerol. Spectra are of diluted enzyme as isolated from the nickel column (purple) and upon addition of one (dark blue), two (light blue), and three (green) equivalents of hemin (B) Difference between UV-Vis spectrum of isolated enzyme and reduced, CO-bound form.

**Figure S2: Conversion of select substrates by PbdAB to their *O*-demethylated products.** PbdAB (1 µM) was incubated for 45 s at 25 °C with 350 µM NADH and 200 µM of (A) *p*-MBA, (B) veratrate, and (C) isovanillate in air saturated 20 mM MOPS, pH 7.2, *I* = 0.1 M containing 1000 U/mL of catalase. Acid-quenched reactions were run on HPLC and compared to chromatograms of authentic standards diluted to 100 µM in the reaction buffer. Aromatics were detected at 280 nm.

**Figure S3: Coupling of NADH oxidation and *p-*hydroxybenzoate production in PbdAB.** Dependence of PbdAB coupling efficiency on (A) ratio of P450 to reductase and (B) ionic strength. NADH oxidation was measured by absorbance at 340 nm and aromatic concentration was determined by HPLC-UV. Reactions initially contained 1 µM PbdA and variable concentration of PbdB for (A) and 1 µM PbdB in (B), 100 µM *p*-MBA and 350 µM NADH. Reactions were conducted at 25 °C in 20 mM MOPS, pH 7.2 at *I* = 0.1 M for (A) and different ionic strengths for (B).

**Figure S4: Steady-state kinetics of PbdAB for *p*-MBA and select analogs.** Reactions contained 0.25 µM PbdA and PbdB in air-saturated 20 mM MOPS, pH 7.2, *I* = 0.1 M and 350 µM NADH. Reactions were incubated at 25 °C. The curves represent best fits of the Michaelis-Menten equation.

**Figure S5: LC/MS of *p*-EB oxidation products by RHA1**. Resting cells in M9 media were incubated with 1 mM *p-*EB for 4 h. The chromatogram shows extracted ion chromatograms for potential *p-*EB oxidation products. 4-Vinylbenzoate (pink) was identified by comparison to authentic standard. The blue and green chromatograms represent predicted oxidation products.

**Figure S6: Growth of RHA1 on *p*-EB.** Growth of WT in M9G supplemented with up to 5 mM of *p*-EB or 5 mM benzoate. OD_600_ was measured after 48 hours incubation.

**Figure S7:** **Alignment of characterized members of the CYP199A subfamily**. Residues that hydrogen bond to the substrate’s carboxylate in PbdA are in blue font. Residues that interact with the aromatic ring are in pink font. The alanine proposed to modulate veratrate activity is highlighted in yellow. Sequences aligned using Clustal Omega (1).

**Figure S8: Active site of PbdA and CYP199A4 with veratrate.** The active sites of PbdA·veratrate (pink; PBD 9G9S) and CYP199A4·veratrate (blue; PBD 4EGN, (2)). Corresponding residues Ala174 in PbdA) and Val164 in CYP199A4 are labelled.

**TABLES**

**Table S1**: Strains used in this study

| Strain | Description | Source |
| --- | --- | --- |
| *E. coli* BL-21 λ (DE3) | Protein expression | Invitrogen |
| *E. coli* DH5α | DNA propagation | Invitrogen |
| RHA1 | *Rhodococcus jostii* RHA1 | (3) |

**Table S2**: Plasmids used in this study

| Plasmid | Description | Source |
| --- | --- | --- |
| pTipQC1 | Rhodococcal expression vector, chloramphenicol resistance | (4) |
| pET28a | *E. coli* expression vector, kanamycin resistance | Novagen |
| pET28a_*pbdA* | pET28a harboring *pbdA* | TWIST Biosciences |
| pET28a_*pbdA_S87A* | To produce PbdA S87A in *E. coli* | This study |
| pET28a_*pbdA_S237A* | To produce PbdA S237A in *E. coli* | This study |
| pET28a_*pbdA_S87A_S237A* | To produce PbdA S87A/S237A in *E. coli* | This study |
| pET28a_*pbdA_R84M* | To produce PbdA R84M in *E. coli* | This study |
| pET28a_*pbdB* | pET28a harboring *pbdB* | (5) |
| pTipQC1_*pbdB* | pTipQC1 harboring *pbdB* | (5) |

**Table** **S3**: Primers used in this study

| Primer | Description | Sequence |
| --- | --- | --- |
| S87A_fwd | Generate pET28a_*pbdA_S87A* | 5’-GCGACCGCCTGCGATATTATTGG-3’ |
| S87A_rev | Generate pET28a_*pbdA_S87A* | 5’-CATGGCGTTTCATATCTAAAATTA-3’ |
| R84M_phos | Generate pET28a_*pbdA_R84M* | 5’-*AACGCCATGGATGCCGCCTTCAA-3’ |
| S237A_phos | Generate pET28a_*pbdA_S237A* and  pET28a_*pbdA_S87A_S237A* | 5’-*GGTAGTTAGAGCGTTATTGACCG-3’ |

*Phosphorylated nucleotide

**Table S4.** Crystallographic diffraction data and refinement statistics PbdA in complex with substrates. Values shown in parentheses are for the highest-resolution shell.

| Protein | **PdbA·**  ***p*-MBA** | **PbdA·**  ***p-*EB** | **PbdA·**  **veratrate** |
| --- | --- | --- | --- |
| PDB code | 9G9Q | 9G9R | 9G9S |
| **Diffraction Data** |  |  |  |
| Space group | P 1 | P 1 | P 1 |
| Cell dimensions |  |  |  |
| a, b, c (Å) | 52.17, 55.11, 64.26 | 51.75, 54.44, 64.02 | 50.99, 55.36, 64.69 |
| α, β, γ (°) | 99.87, 94.9, 103.24 | 99.54, 95.38, 102.64 | 98.68, 95.00, 99.01 |
| Resolution | 52.59 – 2.03  (2.08 – 2.03) | 52.13 – 1.65  (1.68 -1.65) | 63.54 – 1.85  (1.89 – 1.85) |
| Observed reflections | 153731 (7634) | 241267 (12735) | 180310 (12223) |
| Unique reflections | 43063 (2096) | 77677 (3887) | 57209 (3478) |
| Multiplicity | 3.6 (3.6) | 3.1 (3.3) | 3.2 (3.5) |
| I/σ(I) | 6.1 (0.8) | 6.0 (0.7) | 4.8 (0.9) |
| Completeness (%) | 97.92 (96.46) | 96.9 (05.2) | 97.6 (95.9) |
| R_merge_ | 0.171 (1.553) | 0.108 (1.818) | 0.129 (1.29) |
| CC_1/2_ | 0.990 (0.304) | 0.976 (0.345) | 0.990 (0.412) |
| **Refinement** |  |  |  |
| R_work_ (%) | 0.1641 (0.328) | 0.1941 (0.362) | 0.2078 (0.325) |
| R_free_ (%) | 0.2575 (0.395) | 0.2467 (0.363) | 0.2580 (0.336) |
| No. of non-hydrogen protein atoms | 6014 | 6123 | 6044 |
| No. of ligand atoms | 259 | 256 | 218 |
| No. of water molecules | 360 | 454 | 488 |
| Average B-factor (Å^2^) | 23.60 | 25.69 | 24.94 |
| RMS Bonds (Å) | 0.0129 | 0.0087 | 0.0077 |
| RMS Angles (°) | 2.202 | 1.705 | 1.697 |
| **Ramachandran** |  |  |  |
| Preferred (%) | 97.2 | 96.25 | 96.7 |
| Allowed (%) | 2.7 | 3.5 | 3.0 |
| Outliers (%) | 0.1 | 0.25 | 0.01 |
| **Rotamer** **outliers** | 4.7 | 2.5 | 2.7 |

**Table** **S5**: Coupling of NADH oxidation and aromatic substrate turnover of PbdA variants^a^

| **Substrate** | **Variant** | **NADH depletion rate** | **Rate of Aromatic turnover** | **Coupling** |
| --- | --- | --- | --- | --- |
|  |  | **U mg P450^-1^** | **U mg P450^-1^** | **%** |
| *p*-methoxybenzoate | WT | 2700 (100) | 2700 (200) | 100 (3) |
|  | R84M | 1700 (200) | 1100 (200) | 70 (9) |
|  | S87A | 1700 (200) | 800 (200) | 45 (7) |
|  | S237A | 3600 (600) | 2600 (400) | 80 (2) |
|  | S87A/S237A | 400 (30) | 170 (10) | 40 (2) |
| *p*-methoxybenzaldehyde | WT | 140 (30) | N.D. | - |
|  | R84M | 170 (10) | N.D. | - |
|  | S87A | 290 (20) | 80 (30) | 27 (7) |
|  | S237A | 300 (50) | 30 (3) | 9 (3) |
|  | S87A/S237A | 200 (4) | 180 (40) | 90 (20) |
| *p*-methoxyacetophenone | WT | 117 (6) | N.D. | - |
|  | R84M | 170 (30) | N.D. | - |
|  | S87A | 265 (7) | N.D. | - |
|  | S237A | 220 (6) | N.D. | - |
|  | S87A/S237A | 160 (30) | N.D. | - |

^a^Experiments were performed using air-saturated MOPS (*I* = 0.1 M), pH 7.2, at 25 °C. The rate of aromatic substrate turnover was based on the appearance of *O*-demethylated product. Standard deviation is shown in parentheses.

**Table** **S6**: Thermodynamics *of* p-MBA binding to PbdA

| **Variant** | **K_D_** | **ΔG°** | **ΔΔG° ^a^** |
| --- | --- | --- | --- |
|  | **µM** | **kcal/mol** | **kcal/mol** |
| R84M | 36 (6) | -6.1 | 1.3 |
| S87A | 50 (10) | -5.9 | 1.5 |
| S237A | 29 (5) | -6.2 | 1.2 |
| S87A/S237A | 70 (10) | -5.7 | 1.7 |

^a^Comparison of ΔG° of variant to wild type

**References**

1. Sievers, F., Wilm, A., Dineen, D., Gibson, T. J., Karplus, K., Li, W., Lopez, R., McWilliam, H., Remmert, M., Söding, J., Thompson, J. D., and Higgins, D. G. (2011) Fast, scalable generation of high-quality protein multiple sequence alignments using Clustal Omega. *Mol Syst Biol* **7**, 539

2. Bell, S. G., Zhou, R., Yang, W., Tan, A. B., Gentleman, A. S., Wong, L. L., and Zhou, W. (2012) Investigation of the substrate range of CYP199A4: modification of the partition between hydroxylation and desaturation activities by substrate and protein engineering. *Chemistry* **18**, 16677-16688

3. Seto, M., Kimbara, K., Shimura, M., Hatta, T., Fukuda, M., and Yano, K. (1995) A Novel Transformation of Polychlorinated-Biphenyls by *Rhodococcus* Sp Strain RHA1. *Appl Environ Microb* **61**, 3353-3358

4. Nakashima, N., and Tamura, T. (2004) Isolation and characterization of a rolling-circle-type plasmid from *Rhodococcus erythropolis* and application of the plasmid to multiple-recombinant-protein expression. *Appl Environ Microbiol* **70**, 5557-5568

5. Wolf, M. E., and Eltis, L. D. (2024) Preparation of reductases for multicomponent oxygenases. In *Methods in Enzymology*, Academic Press. **703**, pp. 65-85
